# Species Distribution, Antifungal Susceptibility, and Molecular Epidemiology of *Candida* Species Causing Candidemia in a Tertiary Care Hospital in Bangkok, Thailand

**DOI:** 10.3390/jof7070577

**Published:** 2021-07-19

**Authors:** Siriphan Boonsilp, Anchalee Homkaew, Uraporn Phumisantiphong, Daranee Nutalai, Thanwa Wongsuk

**Affiliations:** 1 Department of Clinical Pathology, Faculty of Medicine Vajira Hospital, Navamindradhiraj University, Bangkok 10300, Thailand; uraporn@nmu.ac.th; 2 Division of Central Laboratory and Blood Bank, Faculty of Medicine Vajira Hospital, Navamindradhiraj University, Bangkok 10300, Thailand; anchalee.h@nmu.ac.th (A.H.); daranee.nu@nmu.ac.th (D.N.)

**Keywords:** *Candida*, genotyping, susceptibility testing, goeBURST, multilocus sequence typing

## Abstract

*Candida* species represent a common cause of bloodstream infection (BSI). Given the emergence of non-albicans *Candida* (NAC) associated with treatment failure, investigations into the species distribution, fungal susceptibility profile, and molecular epidemiology of pathogens are necessary to optimize the treatment of candidemia and explore the transmission of drug resistance for control management. This study evaluated the prevalence, antifungal susceptibility, and molecular characteristics of *Candida* species causing BSI in a tertiary-level hospital in Bangkok, Thailand. In total, 54 *Candida* isolates were recovered from 49 patients with candidemia. *C. tropicalis* was the most prevalent species (33.3%), followed by *C. albicans* (29.6%). Most *Candida* species were susceptible to various antifungal agents, excluding *C. glabrata* and *C. tropicalis*, which had increased rates of non-susceptibility to azoles. Most *C. glabrata* isolates were non-susceptible to echinocandins, especially caspofungin. The population structure of *C. albicans* was highly diverse, with clade 17 predominance. GoeBURST analysis of *C. tropicalis* revealed associations between genotype and fluconazole resistance in a particular clonal complex. The population structure of *C. glabrata* appeared to have a low level of genetic diversity in MLST loci. Collectively, these data might provide a fundamental database contributing to the development of novel antifungal agents and diagnostic tests.

## 1. Introduction

*Candida* species are important components of the microflora of the human skin, oral and vaginal mucosa, and gastrointestinal tract. They cause mild superficial and serious infections in humans [1]. *Candida* species represent the fourth most common cause of bloodstream infection (BSI) [2,3]. The incidence of BSI by *Candida* species has increased significantly over the past decade, and *Candida* has become an important cause of nosocomial infections. The risk factors for *Candida* BSI include long-term intensive care unit (ICU) hospitalization, central venous catheterization, organ transplantation, recent broad-spectrum antibiotic use, and immunodeficiency [4]. Virulence factors such as germ tube formation, adhesins, phenotypic switching, biofilm formation, and hydrolytic enzyme production contribute to the pathogenesis of candidiasis [5]. Although *C. albicans* is the main global cause of BSI, non-albicans *Candida* (NAC) species are of increasing concern in patients with hematological conditions, those who underwent organ transplantation, and those admitted to an ICU [6]. The emergence of NAC is a serious issue because NAC species often display intrinsic and/or acquired resistance to commonly used antifungal drugs [6,7]. Different NAC species exhibit varying levels of virulence factors and resistance to antifungal drugs. The species distribution and drug resistance rate of *Candida* species vary between and within geographical regions; therefore, information on the species distribution, fungal susceptibility profile, and molecular epidemiology of NAC is necessary to optimize the treatment of candidemia and explore the transmission of drug resistance for control management. The multilocus sequence typing (MLST) technique is a widely used molecular typing method in molecular epidemiology for pathogenic *Candida* species including *C. albicans*, *C. tropicalis*, and *C. glabrata*. This method is based on the analysis of single-nucleotide polymorphisms in housekeeping gene fragments. The strain databases of different laboratories and different geographical regions can be compared through the online MLST database. This method provides insights into different geographical sources, anatomical sources, drug resistance acquisition transmission, and genetic variation patterns.

In this study, we examined the prevalence and MLST genotype data of *Candida* isolates recovered from patients with BSI at a tertiary care hospital in Bangkok, Thailand, in 2018 and 2019. Antifungal susceptibilities and genetic diversity are also reported. The relationship between susceptibility to azoles and diploid sequence types (DSTs) was also explored.

## 2. Materials and Methods

### 2.1. Yeast Isolates, Identification, and Ethical Statement

In total, 151 blood samples from 49 patients were cultured between June 2018 and July 2019 at the Department of Central Laboratory and Blood Bank, Faculty of Medicine, Vajira Hospital, Navamindradhiraj University (Bangkok, Thailand). Blood samples were incubated using the BACTEC 9240 system (Becton Dickinson, Le Pont de Claix, France). All isolates were identified using matrix-assisted laser desorption/ionization time-of-flight mass spectrometry (Bruker Microflex, Bremen, Germany). All *Candida*-positive blood cultures were incubated overnight on Sabouraud dextrose agar at 37 °C. Yeast species identification was confirmed using the sequence of the internal transcribed spacer sequence (ITS). Amplification and sequencing of the ITS region were performed using the primers ITS5 and ITS4 (Table S1) [8]. This work was approved by The Ethics Committee of the Faculty of Medicine Vajira Hospital, Navamindradhiraj University (COA 61/2561).

### 2.2. Antifungal Susceptibility Testing

Forty-one yeast isolates were available for antifungal susceptibility testing, which was performed using a colorimetric microdilution panel (SENSITITRE YeastOne Trek Diagnostic Systems, Cleveland, OH, USA) containing serial 2-fold dilutions of anidulafungin (0.015–8 μg/mL), micafungin (0.008–8 μg/mL), caspofungin (0.008–8 μg/mL), 5-flucytosine (0.06–64 μg/mL), posaconazole (0.008–8 μg/mL), voriconazole (0.008–8 μg/mL), itraconazole (0.015–16 μg/mL), fluconazole (0.12–256 μg/mL), and amphotericin B (0.12–8 μg/mL). Antifungal susceptibility testing was performed according to the manufacturer’s recommendations. *C. parapsilosis* ATCC 22019 and *C. krusei* ATCC 6258 were used as reference strains for quality control. Minimum inhibitory concentrations (MICs) were measured after 24 h of incubation at 37 °C. Yeast growth was evidenced by a color change from blue (negative, indicating no growth) to red (positive, indicating growth).

### 2.3. DNA Extraction

Genomic DNA was extracted using 10% Chelex solution in 0.1% Triton X-100 and 10 mM Tris buffer (pH 8.0). Then, 200 μL of 10% Chelex solution was added to each yeast cell pellet. The mixture was vortexed for 10 s, heated at 95 °C for 20 min, and centrifuged at 10,000× *g* for 20 s. The supernatant was used as the substrate for PCR.

### 2.4. Genotyping

Yeast isolates were genotyped via MLST. Three *Candida* MLST schemes published for *C. albicans* [9], *C. tropicalis* [10], and *C. glabrata* [11] were used to design the allelic profiles and sequence types (STs). Seven gene fragments were used for *C. albicans: AAT1a*, *ACC1*, *ADP1*, *PMI1b*, *SYA1*, *VS13*, and *ZWF1a*. The markers used for *C. tropicalis* were *ICL1*, *MDR1*, *SAPT2*, *SAPT4*, *XYR1*, and *ZWF1a* [10], and *FKS*, *LEU2*, *NMT1*, *TRP1*, *UGP1*, and *URA3* were used for *C. glabrata* [11]. The gene fragments were amplified by PCR using primers from standardized MLST schemes. PCR was performed using Go Taq^®^ Green Master Mix (Promega, Madison, WI, USA) in accordance with the manufacturer’s instructions. The primers used for amplification of a fragment gene are listed in Table S1. PCR was performed in a reaction volume of 50 μL under the following conditions: initial denaturation at 95°C for 5 min; 35 cycles of denaturation (95 °C for 30 s), annealing (50–60 °C for 30 s), and extension (72 °C for 1 min); and a final extension step at 72 °C for 5 min. All DNA fragments were sequenced in both directions using amplification primers from Macrogen Inc. DNASTAR Lasergene was used to align and edit DNA sequences. Heterozygosity was identified by the presence of two peaks at the same locus on both strands, and degenerated base sequences were recorded according to International Union of Pure and Applied Chemistry recommendations. The consensus sequences of seven loci were determined for all isolates. The consensus sequences of each locus of all isolates were submitted to the MLST database to define the allele profile and DST. The alignment and phylogenetic tree of the sequence data were adjusted manually for each gene using MEGA-X. Population structures were analyzed by the goeBURST algorithm using PHYLOViZ software.

## 3. Results

### 3.1. Candida Species Distribution among Patients with Candidemia

*Candida* species were recovered from all samples collected during the study period. The patient cohort included 29 male (59.18%) and 20 female patients (40.82%). The mean and median patient ages were 57.87 and 66 years, respectively (range, 0.03–99 years). The mortality rate of the study cohort was 53.06%. In total, 54 isolates were recovered from the patients. The most commonly isolated *Candida* species was *C. tropicalis* (33.33%), followed by *C. albicans* (29.63%), *C. glabrata* (22.22%), *C. parapsilosis* (9.26%), *C. nivariensis* (1.85%), *C. guilliermondii* (1.85%), and *C. caribbica* (1.85%). *C. tropicalis* was the most common *Candida* isolate recovered from blood samples (Table 1). Five patients had mixed *Candida* infection, including coinfection with *C. tropicalis* and *C. glabrata* in two patients, *C. albicans* and *C. glabrata* in one patient, *C. tropicalis* and *C. albicans* in one patient, and *C. tropicalis* and *C. parapsilosis* in one patient.

**Figure 1:**
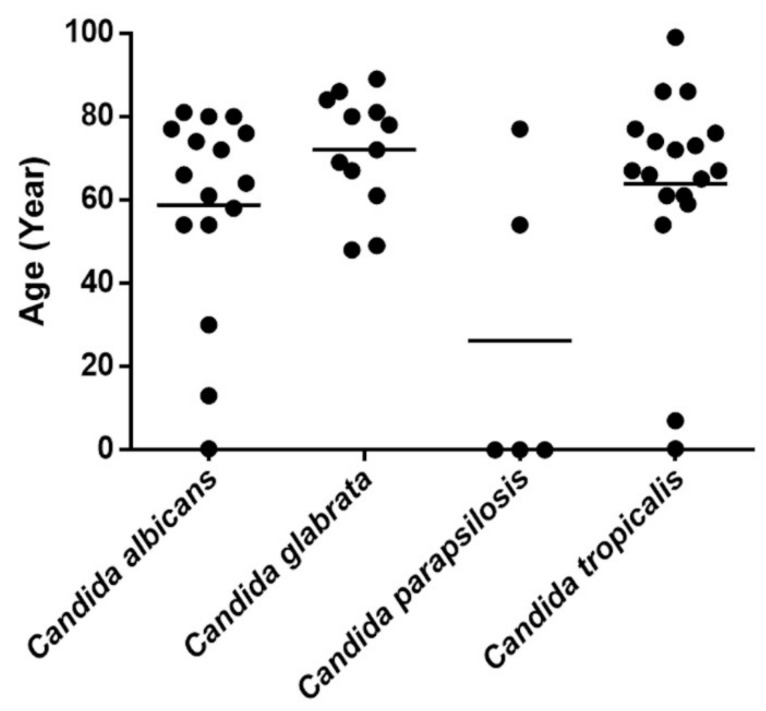
*Candida* species distribution by patient age (years).

*C. tropicalis* was associated with the highest mortality rate (61.11%), followed by *C. albicans* (56.25%), *C. parapsilosis* (40%), and *C. glabrata* (33.33%, Table 2). When analyzing *Candida* species in relation to patient age, we noticed that *C. glabrata* was the most common isolate among elderly patients, whereas *C. parapsilosis* was more common in younger patients. *C. albicans* and *C. tropicalis* were detected in all age groups (Figure 1). Regarding age-specific mortality, the highest mortality rate was recorded among patients aged 61–80 years (31.63%), followed by those aged ≥81 years (10.20%). Patients younger than 60 years had lower mortality rates, including rates of 4.08, 2.04, and 4.98% among patients aged 0–20, 20–40, and 41–60 years old, respectively (Figure S1).

The distribution of *Candida* species isolates by hospital ward was determined. Among the 54 isolates, 38.89, 33.34, 12.96, 11.11, and 3.70% were collected from patients in the surgery ward, internal medicine ward, pediatric/neonatal ICU, emergency room/emergency and medical ICU, and pediatric ward, respectively. *C. albicans* and *C. tropicalis* were found in patients admitted to all wards. *C. albicans* infection was more common in patients admitted to the surgery ward. *C. tropicalis* was a predominant species found in the internal medicine ward. *C. glabrata* was the most common species found in the emergency room/emergency and medical ICU, whereas *C. parapsilosis* was commonly found in the pediatric/neonatal ICU (Figure S2).

### 3.2. Antifungal Susceptibility Testing

Antifungal susceptibility was tested in 41 *Candida* isolates from patients with candidemia using interpretative breakpoints defined by The Clinical and Laboratory Standards Institute. The susceptibility rates of *Candida* against nine antifungal agents are presented in Table 2. *C. albicans* and *C. parapsilosis* were susceptible (wild-type) to all antifungal drugs tested, excluding intermediate susceptibility (50%) to fluconazole in *C. parapsilosis*. The rates of susceptibility to fluconazole were 80, 64.29, and 50% in *C. glabrata*, *C. tropicalis*, and *C. parapsilosis*, respectively. In total, 28.57% of *C. tropicalis* isolates and 20% of *C. glabrata* isolates were resistant to fluconazole. All fluconazole-resistant isolates of *C. tropicalis* and *C. glabrata* were cross-resistant to voriconazole and posaconazole. All *C. tropicalis* isolates were susceptible to echinocandins (anidulafungin, micafungin, and caspofungin). All *C. glabrata* isolates were susceptible to micafungin, whereas 20% and 80% of *C. glabrata* isolates had reduced susceptibility to anidulafungin and caspofungin, respectively. Applying the epidemiological cutoff value, all isolates in this study were susceptible to amphotericin B and 5-flucytosine. The non-wild-type phenotype rates regarding posaconazole susceptibility were 85.71% for *C. tropicalis*, and 90% for *C. glabrata*. Meanwhile, 28.57% of *C. tropicalis* isolates had a non-wild-type phenotype for itraconazole, whereas *C. glabrata* isolates were susceptible to itraconazole. Cross-resistance to voriconazole and posaconazole was observed in 20% of fluconazole-resistant *C. glabrata* isolates. Cross-resistance to voriconazole, itraconazole, and posaconazole was observed in 28.57% of fluconazole-resistant *C. tropicalis* isolates. *C. guilliermondii* was susceptible to micafungin and caspofungin, but it displayed reduced susceptibility to anidulafungin and posaconazole. *C. caribbica* exhibited a high MIC for fluconazole (8 μg/mL).

### 3.3. MLST of C. tropicalis

In total, 14 *C. tropicalis* strains isolated from 14 patients were subjected to MLST. Concatenation of six housekeeping genes obtained a dataset of 2677 bp for each isolate, and 12 (0.45%) polymorphic sites were evaluated. The highest number of variable sites was identified for *MDR1* (5, 1.18%), whereas no polymorphic sites were found for *XYR1* among the study isolates. The 14 isolates were classified into 12 distinct DSTs. Among these, 3 DSTs (DST506, DST667, and DST889) obtained from 4 strains belonged to previously described MLST genotypes, whereas 9 DSTs (DST1097–DST1105) obtained from 10 strains were new genotypes that were added to the *C. tropicalis* MLST central database.

**Figure 2:**
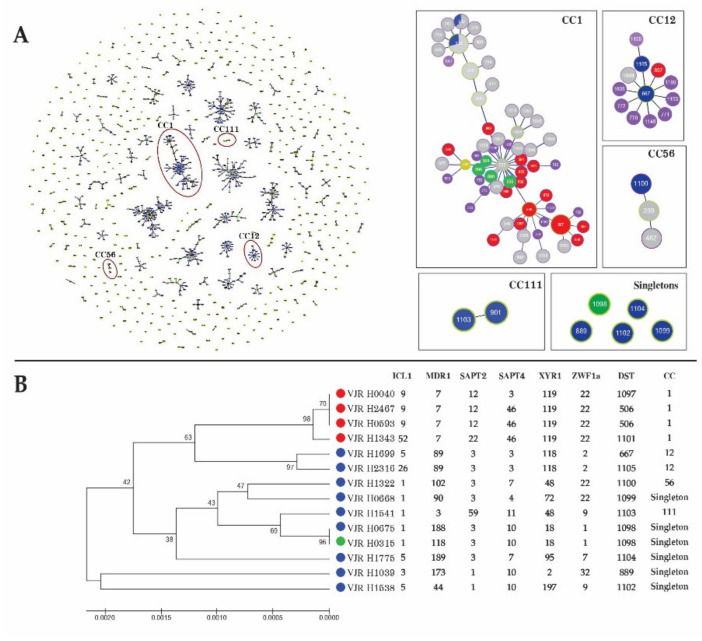
(**A**) Genetic population structure of 1263 *Candida tropicalis* isolates. Population snapshot obtained by goeBURST analysis using the 1161 diploid sequence types (DSTs), which were linked when they had differences in one of the six loci used for multilocus sequence typing (MLST). Single DSTs represent singletons. Clonal complex 1 (CC1; DST506, DST1097, and DST 1101), CC12 (DST667 and DST 1105), CC56 (DST 1100), and CC111 (DST 1103) contain DSTs from this study. DST889, DST1102, DST1104, DST1098, and DST1099 are singletons from this study. The size of each DST reflects the number of associated strains. (**B**) Dendrogram generated from MLST data for 14 *C. tropicalis* isolates from patients with bloodstream infection. Phylogenetic analysis was performed using the unweighted pair group method with arithmetic mean (UPGMA). Strains are classified as fluconazole-susceptible (blue), intermediate (green), and fluconazole-resistant (red). The CCs determined by goeBURST were generally consistent with the groups defined by UPGMA.

Using the goeBURST algorithm, the allelic profiles of these isolates were compared with those of 1263 isolates in the MLST database. Four clonal complexes (CC1, CC12, CC56, and CC111) and five singletons containing the DSTs found in this study are illustrated in Figure 2A. All strains of fluconazole-resistant *C. tropicalis* belonging to three DSTs (DST506, DST 1101, and DST1097) were grouped in cluster CC1. The other 10 isolates were susceptible to fluconazole, and they were scattered among CC12 (DST667 and DST 1105), CC56 (DST 1100), CC111 (DST 1103), and singletons (DST889, DST1102, DST1104, DST1098, and DST1099).

The genetic relationship among the DSTs of *C. tropicalis* isolates in this study was evaluated by constructing an unrooted dendrogram based on MLST data. The unweighted pair group method with arithmetic mean (UPGMA) dendrogram revealed that the 14 isolates belonged to four groups and five singletons. CC1 was the most common cluster type (28.6%), and it was the only fluconazole- and voriconazole-resistant cluster in this study (Figure 2B).

### 3.4. MLST of C. albicans

Ten *C. albicans* isolates from patients with candidemia were subjected to genotyping using the *C. albicans* MLST scheme. The DNA sequences of the fragments of seven different housekeeping genes were concatenated to generate a dataset of 2883 bp for each isolate. MLST revealed that 25 nucleotide sites (0.87%) were variable, and of these, the highest number (*n* = 9) of polymorphic sites was identified for *ADP1*. Conversely, *ACC1* had the lowest number of polymorphic sites (*n* = 2). The allelic diversity of the 10 *C. albicans* isolates regarding the seven loci included nine unique DSTs and one untypable strain. All nine unique DSTs represented novel strains, all of which were submitted to the *C. albicans* MLST scheme (DST3572–DST3580). One isolate was not typable because of two nucleotide insertions in the *VPS13* fragment.

**Figure 3:**
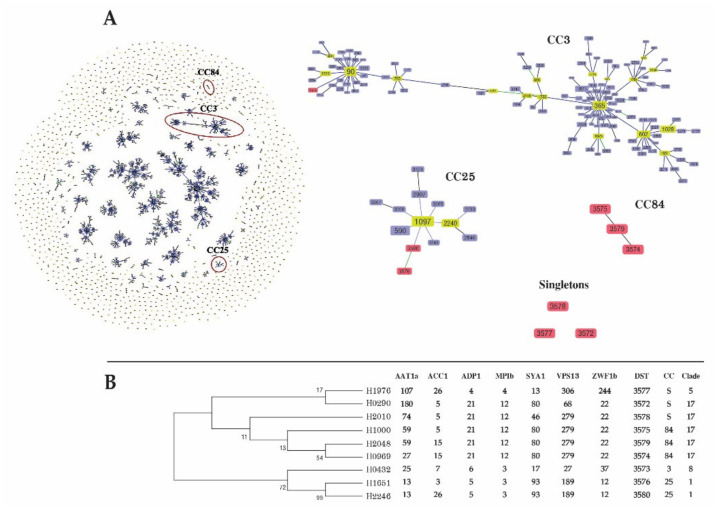
(**A**) goeBURST analysis of 4469 *Candida albicans* entries in the *C. albicans* multilocus sequence typing (MLST) database, which can be grouped into 3637 sequence types. Numbers in nodes represent diploid sequence types (DSTs), light green denotes founder groups, light yellow denotes subgroup founders, and red indicates DSTs identified in the present study. (**B**) Maximum likelihood tree of *C. albicans* based on a concatenated sequence of seven MLST loci among nine *C. albicans* isolates obtained from patients with bloodstream infection in this study.

The goeBURST algorithm was used to analyze the genotype relationships in comparison with the available DSTs (*n* = 3637) in the MLST database. In total, 158 CCs and 1143 singleton strains were identified. Six isolates were placed in three CCs (CC3, CC25, and CC84), whereas three isolates were singletons. Three DSTs (DST3574, DST3575, and DST3579) were grouped in CC84. Two DSTs (DST3576 and DST3580) belonged to CC25. An isolate of DST3573 clustered into CC3. The other three isolates containing DST3572, DST3578, and DST3577 were unrelated singletons (Figure 3). Based on UPGMA, clades 17 (55.6%), 1 (22.2%), 8 (11.1%), and 5 (11.1%) were identified (Figure S3).

### 3.5. MLST of C. glabrata

MLST of 10 *C. glabrata* isolates covered 3345 nucleotides and 31 (0.93%) polymorphic sites. The NMT1 gene featured the highest number (*n* = 10) of polymorphic sites, whereas the URA3 gene carried the lowest number of sites (*n* = 2). The concatenated sequence of the 10 isolates was classified into 4 unique STs. Nine isolates belonged to three previously described STs (ST7, ST55, and ST195). Only one isolate belonged to a new ST (ST199). The data have been submitted to the *C. glabrata* MLST database. The most common DST was ST55 (60%), followed by ST7 (20%), ST195 (10%), and ST199 (10%).

**Figure 4:**
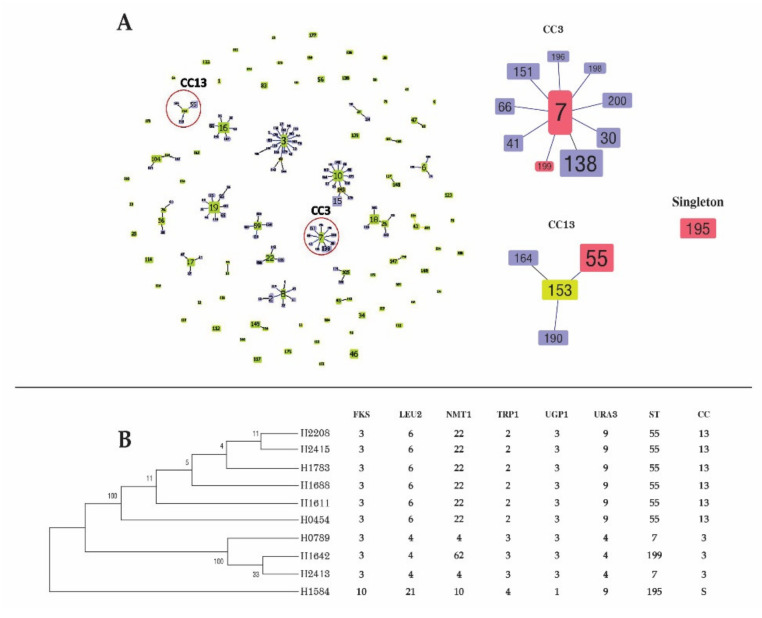
(**A**) goeBURST analysis of 1377 *Candida glabrata* entries in the *C. glabrata* multilocus sequence typing (MLST) database, which could be grouped into 204 sequence types (STs). Numbers in nodes represent STs, light green denotes founder groups, light yellow denotes subgroup founders, and red indicates diploid sequence types identified in the present study. (**B**) Maximum likelihood tree of *C. glabrata* based on the concatenated sequence of 6 MLST loci among 10 *C. glabrata* strains isolated from patients with bloodstream infection in this study.

The goeBURST algorithm was used to analyze the genetic relationships among the 10 *C. glabrata* isolates and 1377 isolates deposited in the *C. glabrata* MLST database. In total, 204 DSTs were grouped into 23 CCs and 53 singletons. All CCs containing DSTs identified in this study are presented in Figure 4. The most common genotype (ST55), which contained six isolates from this study, was grouped in CC13. Two ST7 isolates clustered into CC3. A genotype of ST199 belonged to CC3. DST195 could not be assigned to any known CC.

## 4. Discussion

This study identified four different *Candida* species that cause BSI among patients hospitalized in our institution in 2018 and 2019. *C. tropicalis* was the most common cause of candidemia, followed by *C. albicans*, *C. glabrata*, and *C. parapsilosis*. This is consistent with previous studies, including a report that *C. tropicalis* was the most frequent species isolated from blood samples in the same geographic area of Thailand [12,13,14,15]. Although several studies identified *C. albicans* as the most common fungus isolated from blood samples, more recent studies reported a decreasing frequency of *C. albicans*-associated candidemia and an increasing frequency of candidemia associated with NAC [16,17,18]. Approximately 5.5% of all *Candida* isolates were uncommon species, such as *C. nivariensis*, *C. guilliermondii*, and *C. caribbica*. These rare yeasts have been reported as emerging causes of BSI [19,20,21].

The mortality rate of candidemia in our study was 53.6%, which is comparable to that of previous reports [14,17,22]. The high rate of mortality among elderly patients is consistent with previous findings [23,24]. Moreover, *C. tropicalis* candidemia carried the highest risk of mortality in our study. In line with our findings, several previous studies also described that patients with *C. tropicalis* fungemia have a higher mortality rate than those infected by other *Candida* species [25,26]. Several virulence mechanisms of *C. tropicalis* have been proposed, such as adhesion, biofilm formation, dissemination, hyphae formation, and enzyme production [27]. Therefore, physicians should pay careful attention to patients with persistent candidemia caused by *C. tropicalis*. In the present study, *C. glabrata* was more commonly isolated from elderly patients. This finding is consistent with previous findings of *C. glabrata* as the most prevalent *Candida* species in elderly patients. The mean age of patients with *C. glabrata*-associated candidemia was 72 ± 13.717 years in our study, in line with prior findings [28,29]. This association may be related to earlier treatment with antifungal drugs or underlying diseases. A higher rate of oropharyngeal colonization by *C. glabrata* in older patients has been reported [30]. Patients with *C. parapsilosis*-associated candidemia tended to be younger, which may reflect the use of intravascular devices to treat neonates [31].

Several classes of antifungal drugs such as azoles, echinocandins, and polyenes are available for candidemia treatment. The local epidemiology is an important factor for selecting an antifungal drug. Knowledge of antifungal susceptibility patterns and resistance rates is helpful for patient management in each region, especially in settings with an emerging NAC predominance. In the present study, fluconazole was active against all *C. albicans* isolates, implying that fluconazole could be used as a first-line agent in patients infected by *C. albicans*. Fluconazole resistance was found in *C. tropicalis* and *C. glabrata* isolates, whereas some strains exhibited intermediate susceptibility to fluconazole. This is consistent with recent findings in Asia that fluconazole susceptibility was almost universal for *C. albicans*, whereas NAC strains that cause candidemia had reduced susceptibility [15,32]. In previous studies, low rates of amphotericin B resistance were reported in *C. albicans*, *C. glabrata*, *C. parapsilosis*, *C. tropicalis*, *C. kefyr*, and *C. krusei* isolates [33,34,35]. In our study, amphotericin B displayed excellent activity against all *Candida* species. Overall, itraconazole was extremely active against most *Candida* species, excluding *C. tropicalis*. Many studies reported that non-susceptibility to itraconazole was most common among *C. glabrata* isolates [36,37]. By contrast, no isolates with reduced itraconazole susceptibility were identified in this study. Posaconazole is known to be active against a variety of *Candida* species. In our study, all *C. albicans* and *C. parapsilosis* isolates were susceptible to posaconazole, but 90% of *C. glabrata* and 85.7% of *C. tropicalis* isolates exhibited diminished susceptibility. Resistance to voriconazole was observed in some *C. tropicalis* strains, and most *C. glabrata* isolates displayed reduced susceptibility to voriconazole. Cross-resistance to voriconazole and other azoles such as fluconazole, itraconazole, and posaconazole was also observed in this study. These findings are consistent with previous reports that *C. glabrata* and *C. tropicalis* display higher rates of azole resistance than other *Candida* clinical isolates and exhibit intrinsically lower susceptibility to the azole class [38,39,40,41]. Echinocandins such as micafungin, anidulafungin, and caspofungin represent the preferred choice of treatment for several forms of candidiasis. In our study, the in vitro activity of echinocandins against most *Candida* species was high, excluding *C. glabrata*. These data are consistent with previous results [42,43,44], and they document the excellent potency and spectrum of echinocandins against most *Candida* species other than *C. glabrata*, which has reduced susceptibility in some settings [45].

In line with its higher rate of fluconazole resistance, *C. tropicalis* was associated with the highest mortality rate. Based on its high prevalence and fluconazole resistance, *C. tropicalis* is a serious pathogen reducing the efficacy of fluconazole therapy. MLST can clarify the genetic diversity among isolates and characterize the fluconazole susceptibility pattern [46]. The genetic diversity observed in this study was comparable to that of a previous study in Thailand [12] but higher than that in other countries [47]. CC1, a major cluster associated with azole resistance in this study, is a large fluconazole-non-susceptible group found in Taiwan and China [46,48]. This suggests that CC1 reflects the clonal distribution of azole-resistant isolates across Asia. MLST is suitable for studying the epidemiology of fluconazole-susceptible and fluconazole-resistant *C. tropicalis* clusters.

*C. albicans* is a common cause of candidemia globally. Similarly, *C. albicans* was the second most common cause of candidemia in this study. Globally, antibiotic resistance in *C. albicans* is extremely rare [32,49]. Correspondingly, *C. albicans* blood isolates in our study were highly susceptible to all antifungal agents. This suggests that antifungal agents remain effective in the treatment of *C. albicans* BSI. Globally *C. albicans* remains the most common cause of BSI. The propensity of *C. albicans* to cause infections is the result of genetic plasticity that allows it to adapt to changing or stressful environments. CC84, which was newly identified in this study, reflects the geographical association of genotype clustering in Thailand. The *C. albicans* strains isolated in this study exhibited extremely high genetic diversity. These data confirm previous observations of high genetic diversity among the STs of *C. albicans* [50,51]. This genetic plasticity may be explained by the heterozygosity of the diploid *C. albicans* genome, thereby contributing to a high rate of genetic exchange [52]. Host association is also reported to induce genetic variation in *C. albicans*, which affects virulence phenotypes [53]. Cluster analysis using MLST revealed some phenotypic characteristics that may have a geographic origin [54]. To date, 19 genetic clades have been reported using MLST. In general, clade 1 was most common among *C. albicans* strains causing BSI, whereas clade 17 was rare [50]. Conversely, our result indicates that clade 17 was the most common of the BSI C. albicans, whereas clade 1 was detected less frequently. Clade 17 is uncommon globally among *C. albicans* strains collected from blood samples. A previous study in Thailand reported a high prevalence of clade 17 among Thai patients with *C. albicans* BSI [55]. Clade 17 required stronger hemolytic activity for BSI [56]. This confirms the present observation of the high genetic diversity of clade 17, and its predominance in Thailand.

*C. glabrata* was the third most common cause of candidemia in this study, and it exhibited reduced susceptibility to azoles and echinocandins. Whereas ST55 was the predominant type in this study, a previous study found that ST7 was the major type associated with BSI in Japan, Korea, and China; contrarily, ST55 was uncommon [11,57,58,59]. The prevalence of circulating STs also displayed a geographical bias [60]. Hence, genetic variation among isolates in a particular region cannot be generalized to other areas. We found no association between drug resistance and ST. However, the number of isolates was too small to permit a reliable statistical analysis of isolates or conduct association analysis.

## 5. Conclusions

This study explored the species distribution, molecular epidemiology, and antifungal susceptibility profiles of *Candida* species causing candidemia in our hospital. *C. tropicalis* was the predominant species causing BSI, and strains of this species tended to rapidly acquire resistance to fluconazole. MLST and goeBURST analysis revealed an association between CCs and fluconazole resistance. *C. albicans* was the second most common pathogenic cause, and it was extremely sensitive to various antifungal agents. *C. albicans* strains exhibited high genetic diversity, which allowed them to adapt to new environment conditions. Clade 17 was the predominant *C. albicans* clade causing candidemia. It is possible that this clade possesses higher virulence traits than other isolates. *C. glabrata* exhibited reduced susceptibility to azoles and less genetic diversity. No association existed between drug resistance and ST among *C. glabrata* isolates. This study provides a picture of the molecular epidemiology and drug resistance rates of *Candida* species in Thailand. These data might be helpful for improving therapeutic management of patients with *Candida* infection and boosting the development of novel antifungal agents and diagnostic tests.

## Figures and Tables

**Table 1:** *Candida* isolates among patients with positive hemoculture.

Yeast Isolate	No. of Isolates (%)	Age Mean ± SD	% Mortality
*Candida albicans*	16 (29.63)	58.77 ± 24.37	56.25
*Candida tropicalis*	18 (33.33)	63.90 ± 24.53	61.11
*Candida glabrata*	12 (22.22)	72 ± 13.717	33.33
*Candida parapsilosis*	5 (9.26)	26.23 ± 36.76	40
*Candida nivariensis*	1 (1.85)	54	0
*Candida guilliermondii*	1 (1.85)	0.03	0
*Candida caribbica*	1 (1.85)	59	0

**Table 2:** Minimal inhibitory concentrations (MICs) of *Candida* species from blood as determined using The Clinical and Laboratory Standards Institute breakpoints.

Candida Species	Antifungal Agent	MIC (ug/mL)			Isolates (%)				Isolates (%)	
		Range	MIC_50_	MIC_90_	S	SDD	I	R	wt	Non-wt
*C. albicans*	Anidulafungin	0.015–0.12	0.12	0.12	100	N/A	0	0	0	0
	Micafungin	0.008–0.015	0.008	0.015	100	N/A	0	0	0	0
	Caspofungin	0.03–0.12	0.12	0.12	100	N/A	0	0	0	0
	Flucyctosine	0.06–0.12	0.06	0.12	N/A	N/A	N/A	N/A	N/A	N/A
	Posaconazole	0.03	0.03	0.03	N/A	N/A	N/A	N/A	100	0
	Voriconazole	0.008–0.015	0.008	0.015	100	N/A	0	0	N/A	N/A
	Itaconazole	0.06–0.25	0.12	0.12	N/A	N/A	N/A	N/A	N/A	N/A
	Fluconazole	0.25–1	0.5	1	100	N/A	0	0	N/A	N/A
	AmphotericinB	0.25–0.5	0.5	0.5	N/A	N/A	N/A	N/A	100	0
*C. glabrata*	Anidulafungin	0.03–0.25	0.12	0.25	80	N/A	20	0	N/A	N/A
	Micafungin	0.15–0.03	0.015	0.03	100	N/A	0	0	N/A	N/A
	Caspofungin	0.12–0.25	0.25	0.25	20	N/A	80	0	N/A	N/A
	Flucyctosine	0.06–0.06	0.06	0.06	N/A	N/A	N/A	N/A	N/A	N/A
	Posaconazole	1.0–2.0	2.0	2	N/A	N/A	N/A	N/A	10	90
	Voriconazole	0.25–2	1	2	N/A	N/A	N/A	N/A	10	90
	Itaconazole	0.5–2	1	2	N/A	N/A	N/A	N/A	100	0
	Fluconazole	8–64	32	64	N/A	80	N/A	20	N/A	N/A
	AmphotericinB	0.5–1	0.5	1.0	N/A	N/A	N/A	N/A	100	0
*C. tropicalis*	Anidulafungin	0.12–0.25	0.12	0.12	100	N/A	0	0	N/A	N/A
	Micafungin	0.03–0.06	0.03	0.03	100	N/A	0	0	N/A	N/A
	Caspofungin	0.06–0.25	0.12	0.25	100	N/A	0	0	N/A	N/A
	Flucyctosine	0.06–0.12	0.06	0.06	N/A	N/A	N/A	N/A	N/A	N/A
	Posaconazole	0.12–2	0.25	2	N/A	N/A	N/A	N/A	14.29	85.71
	Voriconazole	0.12–8	0.12	8	57.14	N/A	14.29	28.5	N/A	N/A
	Itaconazole	0.25–4	0.25	2	N/A	N/A	N/A	N/A	71.43	28.57
	Fluconazole	2–256	2	256	64.29	N/A	7.14	28.57	N/A	N/A
	AmphotericinB	0.5–1	1	1	N/A	N/A	N/A	N/A	100	0
*C. parapsilosis*	Anidulafungin	1–2	1	2	100	N/A	0	0	N/A	N/A
	Micafungin	1–2	1	2	100	N/A	0	0	N/A	N/A
	Caspofungin	0.5	0.5	0.5	100	N/A	0	0	N/A	N/A
	Flucyctosine	0.06–0.12	0.12	0.12	N/A	N/A	N/A	N/A	N/A	N/A
	Posaconazole	0.03–0.12	0.06	0.12	N/A	N/A	N/A	N/A	100	0
	Voriconazole	0.015–0.06	0.015	0.12	100	N/A	0	0	N/A	N/A
	Itaconazole	0.06–0.25	0.06	0.25	N/A	N/A	N/A	N/A	N/A	N/A
	Fluconazole	0.5–4	1	2	50	N/A	50	0	N/A	N/A
	AmphotericinB	0.5	0.5	0.5	N/A	N/A	N/A	N/A	100	0

## Data Availability

Sequence typing genotypes are available at PubMLST.org (on date 7 July 2021 for *C. albicans* MLST and 24 August 2021 for *C. tropicalis* MLST).

## Supplementary Materials

Supplementary Materials (Figures S1–S3, Table S1) are available online at https://www.mdpi.com/article/10.3390/jof7070577/s1. Figure S1. Mortality rate by age group among patients with candidemia. Figure S2. The composition of *Candida* species isolated from blood culture in different hospital units. Figure S3. A phylogram was constructed via the unweighted pair group method with arithmetic mean based on the concatenated sequence of seven loci of *Candida albicans*. Clades were assigned by comparing the sequences obtained in this study to the reference sequences. Table S1. Primer sequence and PCR condition.
