# Supplementary material for: Species Distribution, Antifungal Susceptibility, and Molecular Epidemiology of Candida Species Causing Candidemia in a Tertiary Care Hospital in Bangkok, Thailand
Source: J Fungi (Basel). 2021 Jul 19;7(7):577. doi: 10.3390/jof7070577 (PMC8303137; doi:10.3390/jof7070577)
Supplement: Supplementary file 1 [file jof-07-00577-s001.zip › jof-1286570-supplementary.pdf]

Supplementary materials

Figure S1. Mortality rate by age group among patients with candidemia.

Figure S2. The composition of *Candida* species isolated from blood culture in different hospital units.

Figure S3. A phylogram was constructed via the unweighted pair group method with arithmetic mean based on the concatenated sequence of seven loci of *Candida albicans*. Clades were assigned by comparing the sequences obtained in this study to the reference sequences. Each sequence type in this study is represented as a brown dot.

Table S1. Primer sequence and PCR condition

Primer name	Primer Sequence (5′ – 3′)	Product size (bp)	Annealing temperature (°C)	Ref
ITS-4	5′-TCCTCCGCTTATTGATATGC-3′	520-871	55	(8)
ITS-5	5′-GGAAGTAAAAGTCGTAACAAGG-3′			
Candida albicans MLST loci.				
AAT1a-F	5′-ACTCAAGCTAGATTTTTGGC-3′	478	55	(9)
AAT1a-R	5′-CAGCAACATGATTAGCCC-3′			
ACC1-F	5′-GCAAGAGAAATTTTAATTCAATG-3	519	55	(9)
ACC1-R	5′-TTCATCAACATCATCCAAGTG-3′			
ADP1-F	5′-GAGCCAAGTATGAATGATTTG-3′	537	55	(9)
ADP1-R	5′-TTGATCAACAAACCCGATAAT-3′			
MPI b-F	5′-ACCAGAAATGGCCATTGC-3′	486	55	(9)
MPI b-R	5′-GCAGCCATGCATTCAATTAT-3′			
SYA1-F	5′-AGAAGAATTGTTGCTGTTACTG-3′	543	55	(9)
SYA1-R	5′-GTTACCTTTACCACCAGCTTT-3′			
VPS13-F	5′-TCGTTGAGAGATATTCGACTT-3′	741	55	(9)
VPS13-R	5′-ACGGATGGATCTCCAGTCC-3′			
ZWF1b-F	5′-GTTTCATTTGATCCTGAAGC-3′	702	55	(9)
ZWF1b-R	5′-GCCATTGATAAGTACCTGGAT-3′			
Candida glabrata MLST loci.				
FKS-F	5′-GTCAAATGCCACAACAACAACCT-3′	589	55	(11)
FKS-R	5′-AGCACTTCAGCAGCGTCTTCAG -3′			
LEU2-F	5′-TTTCTTGTATCCTCCCATTGTTCA-3′	512	54	(11)
LEU2-R	5′-ATAGGTAAAGGTGGGTTGTGTTGC-3′			
NMT1-F	5′-GCCGGTGTGGTGTTGCCTGCTC-3′	607	59	(11)
NMT1-R	5′-CGTTACTGCGGTGCTCGGTGTCG-3′			
TRP1-F	5′-AATTGTTCCAGCGTTTTTGT-3′	419	50	(11)
TRP1-R	5′-GACCAGTCCAGCTCTTTCAC-3′			
UGP1-F	5′-TTTCAACACCGACAAGGACACAGA-3′	616	57	(11)

UGP1-R	5′-TCGGACTTCACTAGCAGCAAATCA-3′			
URA3-F	5′-AGCGAATTGTTGAAGTTGGTTGA-3′	602	53	(11)
URA3-R	5′-AATTCGGTTGTAAGATGATGTTGC-3′			
Candida tropicalis MLST loci.				
ICL1-F	5′-CAACAGATTGGTTGCCATCAGAGC-3′	737	52	(10)
ICL1-R	5′-CGAAGTCATCAACAGCCAAAGCAG-3′			
MDR1-F	5′-TGTTGGCATTCACCCTTCCT-3′	663	52	(10)
MDR1-R	5′-TGGAGCACCAAACAATGGGA-3′			
SAPT2-F	5′-CAACGATCGTGGTGCTG-3′	658	52	(10)
SAPT2-R	5′-CACTGGTAGCTGAAGGAG-3′			
SAPT4-F	5′-TGCTTCTCCTACAACTTCACCTCC-3′	483	52	(10)
SAPT4-R	5′-ATTCCCATGACTCCCTGAGCAACA-3′			
XYR1-F	5′-AGTTGGTTTCGGATGTTG-3′	479	52	(10)
XYR1-R	5′-TCGTAAATCAAAGCACCAGT-3′			
ZWF1a-F	5′-GGTGCTTCAGGAGATTTAGC-3′	647	52	(10)
ZWF1a-R	5′-ACCTTCAGTACCAAAAGCTTC-3′			
